# Endoscopic fully covered self-expandable metal stent and vacuum-assisted drainage to treat postoperative colorectal cancer anastomotic stenosis with fistula

**DOI:** 10.1007/s00464-022-09831-5

**Published:** 2023-01-23

**Authors:** Shenghe Deng, Ke Liu, Junnan Gu, Yinghao Cao, Fuwei Mao, Yifan Xue, Zhenxing Jiang, Le Qin, Ke Wu, Kailin Cai

**Affiliations:** Department of Gastrointestinal Surgery, Union Hospital, Tongji Medical College, Huazhong University of Science and Technology, Wuhan, 430022 Hubei China

**Keywords:** Endoscopy, Anastomotic complications, Anastomotic stenosis, Anastomotic fistula, Self-expandable metal stent, Homemade vacuum sponge

## Abstract

**Background:**

Digestive tract reconstruction is required after the surgical resection of a colorectal malignant tumor. Some patients may have concomitant anastomotic complications, such as anastomotic stenosis with fistula (ASF), postoperatively. Therefore, we evaluated the efficacy and safety of endoscopic fully covered self-expandable metal stent and homemade vacuum sponge-assisted drainage (FSEM-HVSD) for the treatment of ASF following the radical resection of colorectal cancer.

**Methods:**

Patients treated with FESM-HVSD were prospectively analyzed and followed up for ASF following colorectal cancer treatment in our medical center from 2017 to 2021 for the observation and evaluation of its safety and efficacy.

**Results:**

Fifteen patients with a mean age of 55.80 ± 11.08 years were included. Nine patients (60%) underwent protective ileostomy. All 15 patients were treated with endoscopic FSEM-HVSD. The median time from the index operation to the initiation of FSEM-HVSD was 80 ± 20.34 days in patients who underwent protective ileostomy versus 11.4 ± 4.4 days in those who did not. The average number of endoscopic treatments per patient was 5.70 ± 1.25 times. The mean length of hospital stay was 27.60 ± 4.43 days. FSEM-HVSD treatment was successful in 13 patients, and no patients had any complications. The follow-up time was 1 year. Twelve of 15 (80%) patients achieved prolonged clinical success after FSEM-HVSD treatment, 1 experienced anastomotic tumor recurrence and underwent surgery again, and 1 patient required balloon dilation for anastomotic stenosis recurrence.

**Conclusions:**

FSEM-HVSD is an effective, safe, and minimally invasive treatment for ASF following colorectal cancer treatment. This technique could be the preferred treatment strategy for patients with ASF.

Colorectal cancer (CRC) is among the most frequently diagnosed cancers and a leading cause of cancer-related death worldwide. The incidence of CRC is continuously increasing, and patients are tending to be younger [1–3]. Surgery is the primary treatment for patients with non-metastatic CRC. Because it involves reconstruction of the digestive tract, surgery for CRC is associated with multiple types of postoperative anastomotic complications that are characterized by high morbidity and mortality and can increase the risk of postoperative recurrence and negatively affect long-term prognosis [4]. Common postoperative anastomotic complications include anastomotic bleeding, fistula, leakage, and stenosis, with varying incidence of 1.8–19.8% [5, 6]. The incidence of anastomotic fistula and anastomotic stenosis are reportedly 1.8–10.4% and 2.5–19.5%, respectively [7, 8].

Treatment options for postoperative anastomotic complications are surgical or conservative. With technological advances, endoscopic surgery is being increasingly used, primarily including endoscopic metal clipping, self-expandable metallic stent (SEMS) placement, fibrin glue injection, and endoscopic vacuum-assisted therapy (EVT) [9, 10]. The endoscopic treatment of anastomotic stenotic complications mainly includes temporary SEMS placement, endoscopic incision, and balloon dilatation [11]. Moreover, endoscopy is an increasingly popular minimally invasive technique for the treatment of anastomotic complications.

However, the effectiveness of single endoscopic treatment is often unsatisfactory, possibly owing to the complexity of anastomotic complications such as anastomotic stenosis with fistula (ASF) [12] and anastomotic obstruction complicated by multiple fistula [13] as well as the potential requirement for multiple treatments of varying therapeutic effects. No large-scale study has validated the appropriateness of the current treatment guidelines for the treatment of this rare and complex anastomotic complication. Therefore, safe, effective, and minimally invasive first-line treatment regimens are needed.

In our medical center, based on minimally invasive treatment for CRC, we adopted endoscopic technology to treat postoperative anastomotic complications. This study evaluated the efficacy and safety of endoscopic FSEM-HVSD in the treatment of postoperative ASF after the radical resection of CRC.

## Materials and methods

### Data source

Patients who underwent FSEM-HVSD treatment for postoperative anastomotic stenosis with fistula in the Department of Gastrointestinal Surgery of Wuhan Union Hospital in 2017–2021 were prospectively analyzed and followed up. The study protocol was reviewed and approved by the Ethics Committee and Institutional Review Committee of Wuhan Union Hospital (No.2018-S377). All patients provided written informed consent, and all procedures were performed in accordance with the Declaration of Helsinki.

### Inclusion and exclusion criteria

Patients diagnosed with ASF after CRC surgery and treated with FSEM-HVSD were enrolled. The inclusion criteria were as follows: (1) anastomotic stenosis concomitant with obvious anastomotic fistula; and (2) occult anastomotic fistula found after treatment for anastomotic stenosis. The exclusion criteria were as follows: (1) abnormal coagulation; (2) severe anastomotic leakage, obvious infection, and unstable vital signs; and (3) refusal to be treated with endoscopic FSEM-HVSD.

### Data collection

The collected patient information included age, sex, body mass index (BMI), time of diagnosis, primary tumor location, neoadjuvant therapy, preventive stoma, anastomotic location (distance from anal margin), stenosis length, anastomotic fistula diameter, number of endoscopic treatments, length of hospital stay, and adverse events during treatment, including high fever, severe bleeding, perforation, and stent displacement.

Technical success was defined as successful FSEM-HVSD treatment at the anastomotic site. Clinical success was defined as symptom relief, unobstructed recovery of stenosis and anastomotic fistula closure without major complications, and no recurrence during follow-up. Early clinical success refers to the absence of recurrence within 1 month from admission to successful treatment. Long-term success was defined as the absence of recurrence or repeated treatment of anastomosis within 1 year of follow-up after successful treatment.

### Homemade vacuum sponge creation

**Fig. 1:**
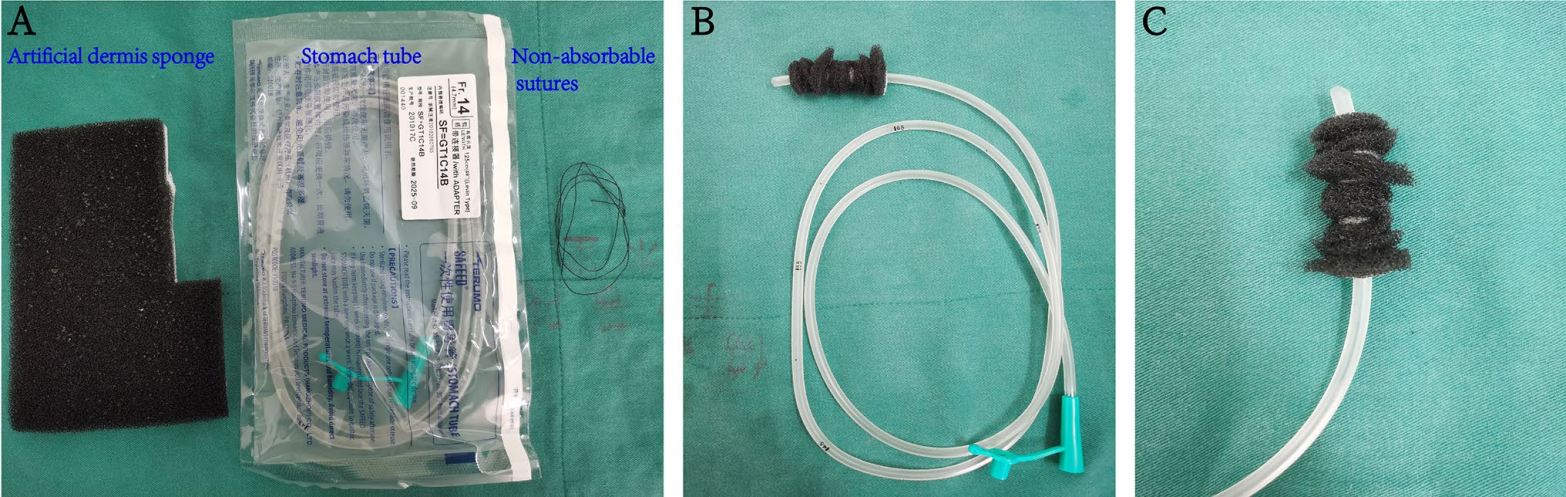
A homemade vacuum sponge material and method. **A** homemade vacuum sponge was created of ordinary stomach tube (14–16 Fr), artificial dermis sponge, and non-absorbable sutures. **B** The overall appearance of homemade vacuum sponge. **C** The head end of a homemade vacuum sponge

The homemade vacuum sponge was created of ordinary stomach tube (14–16 Fr), artificial dermis sponge, and non-absorbable sutures. First, 4–5 side holes were cut 3–4 cm from the end of the stomach tube to facilitate drainage. Second, the size of the anastomotic fistula was evaluated endoscopically, and the artificial dermis sponge that could cover the area of the fistula was cut out. Subsequently, the cut sponge was wrapped around the end of the stomach tube with a side hole and fixed with non-absorbable sutures. Finally, to increase the contact area between the artificial dermis sponge and the anastomotic fistula, an annular incision is made on the fixed artificial dermis sponge at a distance of 3–5 mm. Do not cut through the device. Following its production, the endoscopic implantation was performed (Fig. 1).

### Endoscopic surgical techniques

For the transanal endoscopic examination of the area around the anastomosis, we checked for anastomotic stenosis and anastomotic fistula. After a final diagnosis of ASF was made, the distance between the fistula and the stenosis was first observed, while the space between them was subsequently assessed to check whether a homemade vacuum sponge could be fitted. If the space was adequate, the homemade vacuum sponge was routinely placed at the anastomotic fistula and examined endoscopically. After sponge placement, the guide wire was used to pass through the narrow section of the anastomosis under X-ray monitoring. After the guide wire was passed through the narrow section of the anastomosis, it was fixed and the recyclable fully covered SEMS (MTN-CG-G; Micro-Tech Co., Ltd., Nanjing, China) was released under guide wire and X-ray guidance.

If the space around the anastomotic fistula was too small to accommodate the homemade vacuum sponge, we first placed a fully covered SEMS, identified a space around the anastomotic fistula to place the homemade vacuum sponge, removed the fully covered SEMS, placed the homemade vacuum sponge at the anastomotic fistula, and subsequently re-inserted the fully covered SEMS. The homemade vacuum sponge was replaced once every 5 days, and the fully covered SEMS was removed and preserved. The homemade vacuum sponge drained for 2 days, and the recovered SEMS was subsequently placed in the narrow section of the anastomosis.

**Fig. 2:**
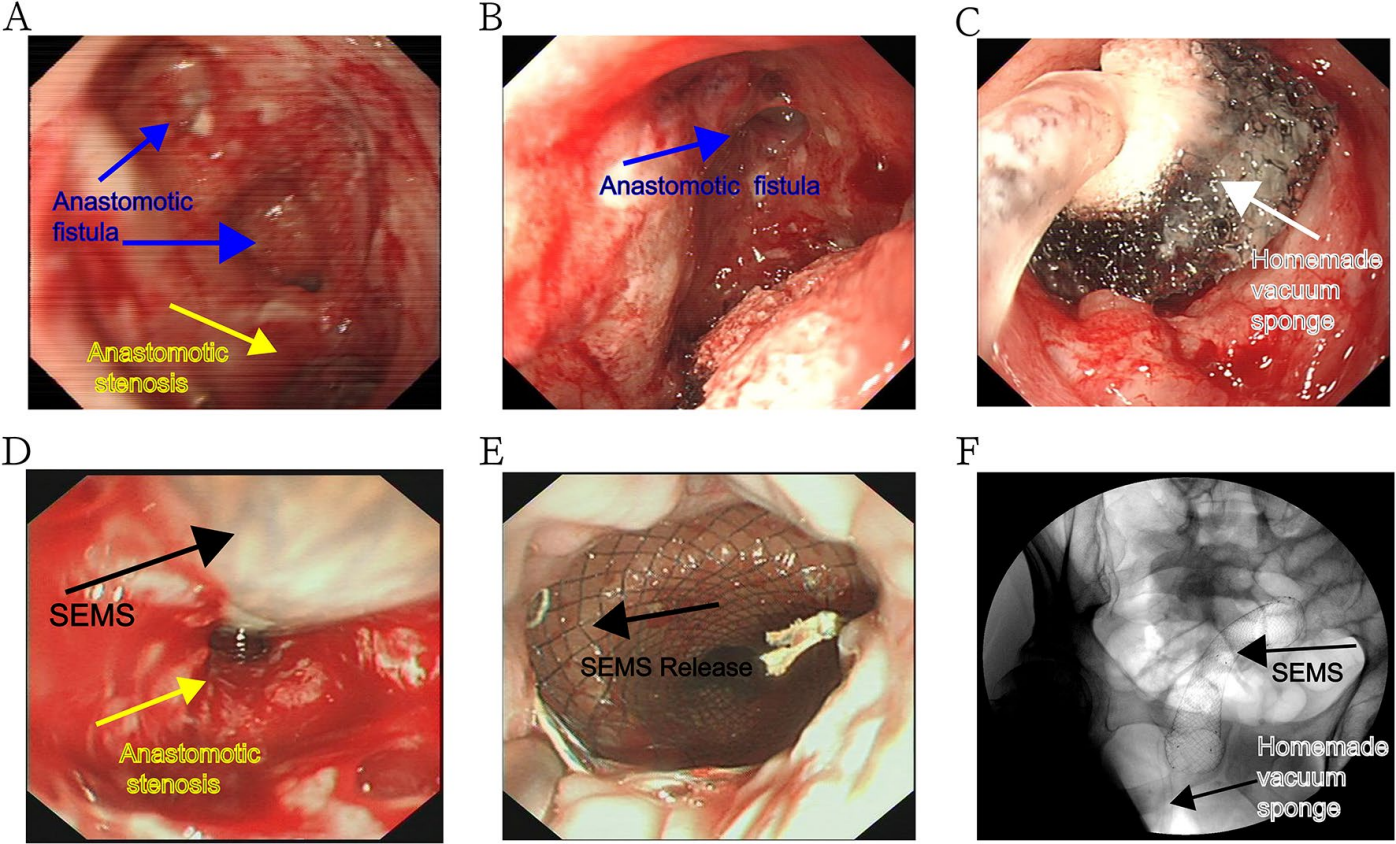
Endoscopic surgical techniques. **A** Anastomotic stenosis with fistula after CRC surgery. **B** Anastomotic fistula and its surrounding tissue. **C** A homemade vacuum sponge was placed at the anastomotic fistula. **D** Anastomotic stenosis was treated with full covered self-expanding metal stent (SEMS). **E** Release the SEMS. **F** X-ray monitor to check whether the homemade vacuum sponge and SEMS were in the correct position

The condition of the anastomosis was inspected regularly, and the diameter of the anastomotic fistula and the stenosis were observed. If the anastomotic fistula was closed, we checked whether the stenosis was relieved. If stenosis persisted, the fully covered SEMS was used further. All procedures were performed by skilled endoscopic surgeons (Fig. 2).

### Treatment outcomes and follow-up

Detailed anastomotic changes were recorded for each patient after each treatment. After successful FSEM-HVSD treatment, all patients were followed up for at least 12 months. Clinic visits were scheduled for 3, 6, and 12 months postoperative to check for recurrence and whether long-term clinical success was achieved.

### Statistical analysis

All statistical calculations were performed using SPSS version 22.0 (SPSS, Chicago, IL, USA). Quantitative variables are described as arithmetic mean and standard deviation, while qualitative variables are expressed as raw numbers, proportions, and percentages.

## Results

### Demographic and clinical data

The 15 included patients included 12 men and 3 women. The mean patient age and BMI were 55.80 ± 11.08 years and 24.68 ± 2.00, respectively. Ten patients underwent radical resection of rectal cancer, 5 underwent radical resection of sigmoid colon cancer, 9 received neoadjuvant chemoradiotherapy, and 10 underwent primary protective ileostomy. The mean distance between ASF and the anal margin was 7.5 ± 2.37 cm; 3 patients had a distance > 10 cm. The mean anastomotic stenosis length was 2.70 ± 0.95 cm, while the mean fistula diameter was 23.20 ± 7.08 mm. The anastomotic fistula diameter was > 30 mm in 4 patients. The median time from the index operation to the initiation of FSEM-HVSD was 80 ± 20.34 days in patients who underwent protective ileostomy and 11.4 ± 4.4 days in those who did not. All 15 patients received FSEM-HVSD treatment (Table 1).

**Table 1:** Clinicopathological characteristics of anastomotic stenosis with fistula

Variables	Cases (*n* = 15)
Age (years)	55.80 ± 11.08
Gender	
Male	12
Female	3
BMI (kg/m^2^)	24.68 ± 2.00
Median time from index operation to the initiation of FSEM-HVSD, (days)	
Patients with ileostomy	80 ± 20.34
Patients without ileostomy	11.4 ± 4.4 day
Location of tumor	
Rectal	10
Sigmoid colon	5
Neoadjuvant therapy	
Yes	9
No	6
Primary protective ileostomy	
Yes	10
No	5
Distance to the anal verge (DAV)(cm)	7.5 ± 2.37
< = 10	12
> 10	3
Length of stricture(cm)	2.70 ± 0.95
Diameter of fistula(mm)	23.20 ± 7.08
< = 30	11
> 30	4

*FSEM-HVSD* Fully covered self-expandable metal stent and homemade vacuum sponge assisted drainage

### FSEM-HVSD treatment outcomes

**Fig. 3:**
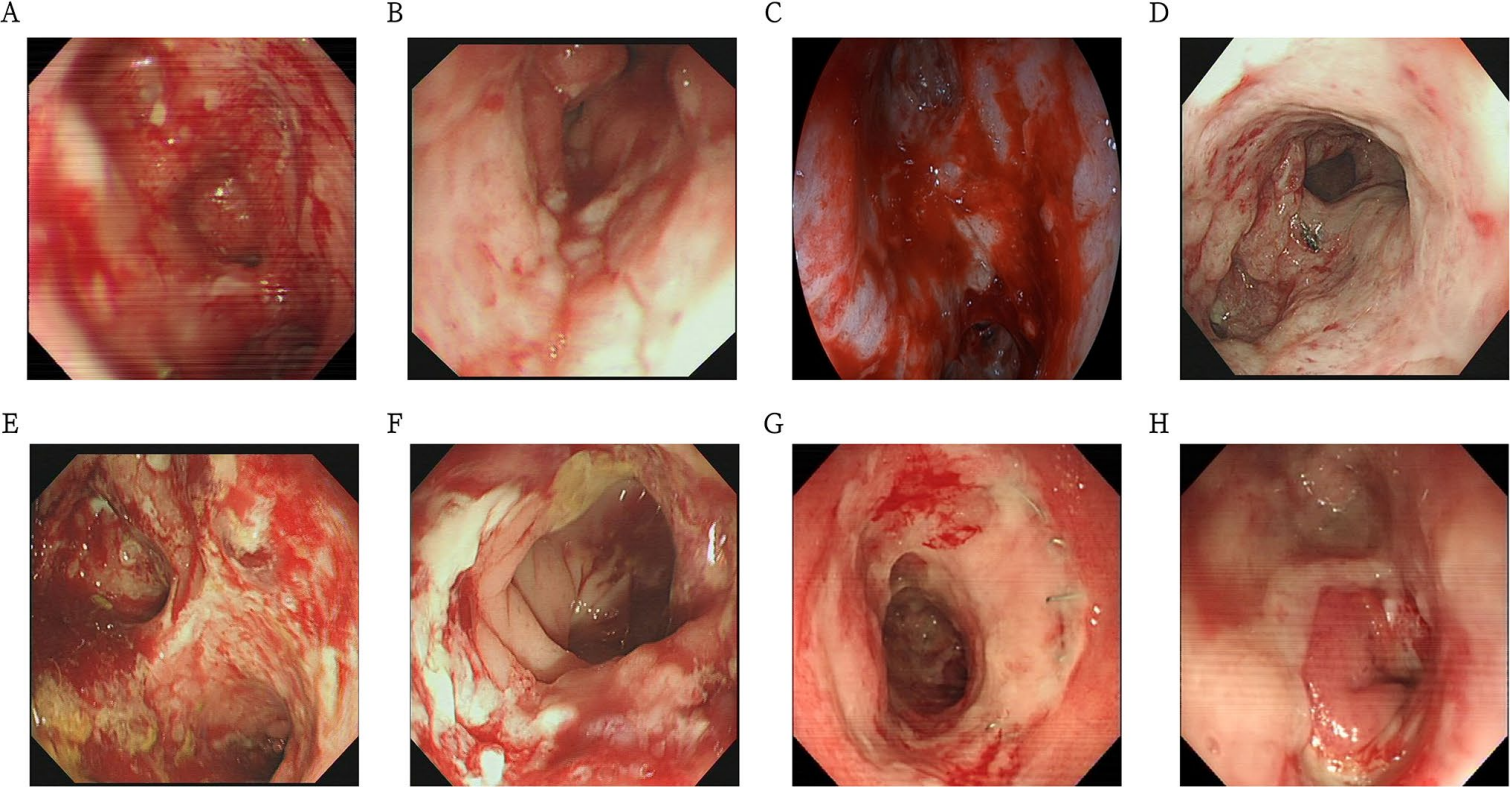
Changes of anastomotic stenosis with fistula in one patient for each endoscopic treatment. **A** Anastomotic stenosis with fistula before treatment. **B** Anastomotic stenosis was slightly improved after the first treatment, but anastomotic fistula was not significantly improved. **C** After the second treatment, inflammatory granulated tissue around the anastomosis was hyperplasia, multiple ulcers were alleviated, anastomotic fistula and anastomotic stenosis were improved. **D** After the third treatment, anastomotic inflammation was relieved, anastomotic fistula was narrowed, and anastomotic stenosis still existed. **E** After the fourth treatment, anastomotic granulated tissue was hyperplasia, fistula was closured, and stenosis was relieved. **F** After the fifth treatment, anastomotic stenosis with fistula was successfully cured. **G–H** Anastomotic stenosis with fistula was cured and didn’t recurred in 1-month (G) and 1-year (H) follow-up

The mean operation time was 27.00 ± 5.87 min. The homemade vacuum sponges were replaced a mean 5.00 ± 0.94 times. The endoscopic treatments were performed a mean 5.70 ± 1.25 times, and the mean total length of hospital stay was 27.60 ± 4.43 days. No patient had complications following endoscopic treatment. All patients treated with FSEM-HVSD were started on an oral liquid diet; those who did not undergo protective ileostomy were started 1–2 days postoperative. Due to mild fever, abnormal drainage, and/or laboratory changes postoperatively (white blood cell count, > 10 × 10^9^/L; procalcitonin, > 1.0 ng/mL; C-reactive protein, > 10 mg/L), 6 of 15 patients (40.00%) required broad-spectrum intravenous antibiotics following treatment (Table 2, Fig. 3).

**Table 2:** Therapeutic effect and follow-up of FSEM-HVSD

Variables	Cases (*n* = 15**)**
Number of endoscopic treatments	5.70 ± 1.25
Endoscopic operation time (min)	27.00 ± 5.87
Number of the endo-sponge used	5.00 ± 0.94
Total length of stay (days)	27.60 ± 4.43
Technical success	15
Clinical success	14
Treatment-related complications	0
Closure of ileostomy time (week)	12.30 ± 4.55
Prolonged clinical success (*n*)	13

*FSEM-HVSD* Fully covered self-expandable metal stent and homemade vacuum sponge assisted drainage

Technical success was achieved in all cases. The early clinical success rate was 93.33% (14/15). For the 5 patients who did not undergo ileostomy during the initial cancer resection, no additional ileostomy was required during FSEM-HVSD treatment. One of fifteen patients (6.67%) abandoned the FSEM-HVSD treatment and finally chose surgical intervention on the 20th day of treatment (Table 2).

### Outcome of follow-up

Patients were followed up for 1 year. During follow-up, anastomotic stenosis recurred in 2 patients. One patient underwent surgical treatment for anastomotic stenosis caused by tumor recurrence. Another required balloon dilation due to recurrence of benign stenosis. In all 10 patients who underwent protective ileostomy, the mean duration between reestablishing continuity of the digestive tract and closing the ileostomy was 12.30 ± 4.55 weeks after the end of endoscopic treatment. Twelve of fifteen (80.00%) patients experienced good long-term results after FSEM-HVSD therapy.

## Discussion

In our study, FSEM-HVSD was used for the first time to treat patients with ASF. Our results suggest that technical success was achieved in all 15 patients and that early clinical success was achieved in 14 (93.33%). During the 1-year follow-up period, anastomotic stenosis recurred in 2 patients, including 1 whose tumor recurred and required surgery. Twelve patients (80.00%) achieved long-term clinical success. Moreover, our proposed technique is simple to perform, with an average endoscopic operation time of 27.00 ± 5.87 min. Simultaneously, our homemade vacuum sponge, which is made of cheap material and uses a recyclable fully covered SEMS, can reduce the financial burden on patients.

In our study, the median time from the index operation to the initiation of FSEM-HVSD was different among patients who did versus did not undergo ileostomy. As for the diagnosis and treatment time of patients with ASF, our literature search revealed no large study to date. Repici et al. [12] reported the case of a 76-year-old woman who developed anastomotic obstruction complicated by multiple fistulas at 1 year after sigmoid resection. Lamazza et al. [13] reported that 10 patients developed simple stenosis at a mean 5 months after colorectal resection and that another 10 patients developed stenosis with leakage at a mean 2 weeks after colorectal resection. All patients had abdominal distension and fever and an increased white blood cell count. Kuhn et al. [14] reported 218 cases of colorectal defects in patients who received EVT with a median time from diagnosis to EVT of 10 (range, 1–91) days. The time to diagnosis differed between medical centers. In fact, for patients with early onset of anastomotic leakage accompanied by corresponding symptoms, including abdominal distension, fever, and/or abnormal laboratory results, early diagnosis and treatment are possible. For patients without obvious symptoms, the time to early diagnosis may need to be extended.

In our study, the time to diagnosis was defined as the time from resection of primary CRC to the first endoscopic treatment. The reason for the relatively long diagnosis time was that most patients underwent ileostomy in the initial operation (10 cases); therefore, the early symptoms of anastomotic stenosis with fistula were not obvious. Patients who wanted to close the ileostomy after 3–6 months or had abdominal discomfort during follow-up and underwent endoscopy to find anastomotic complications had a mean diagnostic time of 80 ± 20.34 days, longer than that of patients in other studies. For the 5 patients who did not undergo ileostomy, postoperative abdominal pain, abnormal drainage fluid, and anastomotic complications occurred. The time to diagnosis of such patients was shorter (11.4 ± 4.4 days), not significantly different from that of patients in other studies.

There are few relevant studies and reports on the treatment of postoperative ASF in patients with CRC. Lamazza et al. [15] reported using metal stents to treat 9 patients with ASF for a mean 4 months, among which 2 patients underwent permanent ileostomy due to persistent fistulas. Moreover, among these 9 patients, 3 experienced stent displacement and 1 experienced recurrent obstruction requiring balloon dilation. Repici et al. [12] reported a successful application of SEMS for the palliative treatment of recurrent malignant colorectal anastomosis complicated by intestinal obstruction and multiple fistulas. The fistula was confirmed as closed and intestinal patency restored. However, the patient died 13 weeks after the endoscopic palliative treatment. Compared with our treatment regimen, FSEM-HVSD has the advantage of a shorter treatment period, fewer endoscopic complications, and a higher cure rate than metal stents alone. Moreover, FSEM-HVSD technology can reportedly be applied to treat complex upper gastrointestinal anastomotic fistulas.

The stent-over-sponge approach is a new method for the treatment of complicated anastomotic fistula after esophageal surgery, especially in cases of large anastomotic fistula, difficult stent placement, or ineffective stent treatment [16, 17]. We also found that endoscopic stent combined with our homemade vacuum sponge drainage is a safe and effective strategy for treating postoperative anastomotic stenosis with fistula in patients with CRC.

Currently, there are few studies regarding the application of vacuum sponge drainage in the treatment of anastomotic complications, especially complicated cases. Similar to some studies, we used a homemade vacuum sponge system device to treat postoperative anastomotic complications [18, 19]. In recent years, EVT has been increasingly used in the treatment of anastomotic complications following CRC surgery. The relevant literature is summarized in Table 3. Through a literature review, we found that EVT is mainly used to treat anastomotic fistula with a good treatment effect [14, 18–25]. However, no studies have reported on the treatment of postoperative ASF for CRC. To the best of our knowledge, this study is the first to adopt FSEM-HVSD technology for the treatment of such complications. Meanwhile, the study results show that FSEM-HVSD is a safe and effective minimally invasive treatment strategy for postoperative ASF.

**Table 3:** EVT of CRC anastomotic fistula

Author	Year	Cases	Treatment	Treatment time (days)	Number of sponge insertions	Successful treatment	Treatment failure	Endoscopic surgery complications	Follow-up
Kuehn [18]	2007–2015	20	EVT	23	7	18	2	1 case bleeding	3 cases anastomotic stenosis
Bobkiewicz [19]	2018–2019	6	EVT	20.7 ± 8.8	5.8 ± 2.3	6	0	0	Purulent discharge and defective sizes are reduced
Jagielski [20]	2016–2019	18	Transrectal vacuum therapy	22	6	15	1	2 cases bleeding	2 cases with recurrent pelvic absces
Kühn [21]	2001– ~	20	EVT	21	7	18	2	0	2 cases luminal stenosis
Kühn [14]	2001–2019	281	EVT	25	8	256	25	0	No detailed description
Arezzo [14]	2008–2013	14	EVT	40.5	Unknow	11	4	0	No recurrence
Strangio [22]	2008–2013	25	EVT	28	9	22	3	0	No mortality related to the procedure was observed
Van Koperen [23]	2006–2008	16	EVT	40	13	9	4	1 case bleeding	The stoma had been closed in five patients
Riss [24]	2007–2008	9	EVT	21	Unknow	6	3	1 case died of heart attack	No detailed description
Van Koperen [25]	2007	2	EVT	Unknow	Replace every 3–4 days	2	0	0	No recurrence

*EVT* Endoscopic vacuum-assisted therapy

The principle of FSEM-HVSD is that the metal stent can treat anastomotic stenosis. Simultaneously, some hidden anastomotic fistula may be exposed. Moreover, the EVT was combined to keep the fistula clean, reduce continuous leakage, promote the proliferation of granulation tissue, and close the fistula.

In this study, SEMS was preferred to relieve the obstruction in patients with anastomotic stenosis and fistula. Through a literature review, we learned that endoscopic balloon dilatation is the simplest treatment for anastomotic stenosis, but its recurrence rate is approximately 20% [26]. After balloon dilatation, the maintenance time is short and the expanded scar tissue shrinks easily, requiring multiple balloon dilatations. The advantage of using SEMS is that its expansion is continuous and has a shaping effect, thus achieving a long-term effect. Lamazza et al. [13] reported that, in the case of simple anastomotic stenosis, SEMS have a complementary effect to balloon dilatation, but in the case of anastomotic stenosis with leakage, SEMS is an effective auxiliary tool that can improve the healing rate. Caruso et al. [27] reported that SEMS is a safe and effective treatment for refractory anastomotic colorectal stenosis. Similarly, for patients with anastomotic leakage and stenosis, we believe that stenosis relief is a prerequisite for fistula closure. Therefore, for our patient, combined with knowledge from published case reports [12, 28–30], we tended to use a fully covered SEMS and vacuum sponge-assisted drainage to treat ASF.

For the homemade vacuum sponge, we chose to use medical artificial dermis sponge. For the drainage tube, we chose to use an ordinary gastric tube. After cutting the artificial dermis of the corresponding size according to the actual anastomosis shape, the side hole was made at the end of the gastric tube and placed into the artificial dermis, which was fixed with non-absorbable sutures. Moreover, it is recommended that the medical sponge be as fluffy as possible to increase the contact area with the fistula and speed the healing. According to our treatment experience, the homemade vacuum sponge was changed once a week, and the entire treatment process lasted for 1–2 months. After the homemade vacuum sponge was replaced, EVT treatment was recommended for 2 days, and the metal stent was subsequently placed to prevent intestinal perforation caused by its long-term compression. Furthermore, the homemade vacuum sponge is very inexpensive and does not cause an additional financial burden on patients. Simultaneously, the device can also be used to treat a common anastomotic fistula. Therefore, we believe that our proposed technique may be the preferred treatment strategy for some specific anastomotic complications.

This study has some limitations. First, it was a single-center prospective study that lacked a control group and randomization. Second, the sample size was small, mainly because the incidence of such complications is very low, and a large sample for validation is needed. Nevertheless, this is a rare and significant study, and our results suggest that FSEM-HVSD is a minimally invasive, safe, and effective treatment strategy for postoperative anastomotic stenosis with fistula management in patients with CRC. The diagnosis, treatment, and management of gastrointestinal anastomotic complications remain under study, and we are constantly working on developing safer, more effective, and more minimally invasive techniques to manage postoperative gastrointestinal anastomotic complications.

In conclusion, FSEM-HVSD is an effective, safe, and minimally invasive treatment for ASF following CRC. Although our results confirm the feasibility of this technique, further validation is needed in a large-sample clinical studies.
